# Comparative Genomics Identifies Putative Interspecies Mechanisms Underlying Crbn-Sall4-Linked Thalidomide Embryopathy

**DOI:** 10.3389/fgene.2021.680217

**Published:** 2021-06-23

**Authors:** Thayne Woycinck Kowalski, Gabriela Barreto Caldas-Garcia, Julia do Amaral Gomes, Lucas Rosa Fraga, Lavínia Schuler-Faccini, Mariana Recamonde-Mendoza, Vanessa Rodrigues Paixão-Côrtes, Fernanda Sales Luiz Vianna

**Affiliations:** ^1^Post-Graduation Program in Genetics and Molecular Biology, PPGBM, Universidade Federal do Rio Grande do Sul, UFRGS, Porto Alegre, Brazil; ^2^Laboratory of Medical Genetics and Evolution, Genetics Department, Universidade Federal do Rio Grande do Sul, UFRGS, Porto Alegre, Brazil; ^3^Laboratory of Genomic Medicine, Center of Experimental Research, Hospital de Clínicas de Porto Alegre, HCPA, Porto Alegre, Brazil; ^4^National Institute of Medical Population Genetics, INAGEMP, Porto Alegre, Brazil; ^5^Bioinformatics Core, Hospital de Clínicas de Porto Alegre, HCPA, Porto Alegre, Brazil; ^6^Centro Universitário CESUCA, Cachoeirinha, Brazil; ^7^Post-Graduation Program in Biodiversity and Evolution, PPGBioEvo Institute of Biology, Universidade Federal da Bahia, UFBA, Salvador, Brazil; ^8^Department of Morphological Sciences, Institute of Health Sciences, Universidade Federal do Rio Grande do Sul (UFRGS), Porto Alegre, Brazil; ^9^Post-Graduation Program in Medical Science, Universidade Federal do Rio Grande do Sul, UFRGS, Porto Alegre, Brazil; ^10^Teratogen Information System, SIAT, Medical Genetics Service, Hospital de Clínicas de Porto Alegre, HCPA, Porto Alegre, Brazil; ^11^Institute of Informatics, Universidade Federal do Rio Grande do Sul, UFRGS, Porto Alegre, Brazil

**Keywords:** IMiDs, teratogenesis, teratogens, comparative genomics, co-expression, C2H2, NOS3, synteny

## Abstract

The identification of thalidomide–Cereblon-induced SALL4 degradation has brought new understanding for thalidomide embryopathy (TE) differences across species. Some questions, however, regarding species variability, still remain. The aim of this study was to detect sequence divergences between species, affected or not by TE, and to evaluate the regulated gene co-expression in a murine model. Here, we performed a comparative analysis of proteins experimentally established as affected by thalidomide exposure, evaluating 14 species. The comparative analysis, regarding synteny, neighborhood, and protein conservation, was performed in 42 selected genes. Differential co-expression analysis was performed, using a publicly available assay, GSE61306, which evaluated mouse embryonic stem cells (mESC) exposed to thalidomide. The comparative analyses evidenced 20 genes in the upstream neighborhood of *NOS3*, which are different between the species who develop, or not, the classic TE phenotype. Considering protein sequence alignments, RECQL4, SALL4, CDH5, KDR, and NOS2 proteins had the biggest number of variants reported in unaffected species. In co-expression analysis, *Crbn* was a gene identified as a driver of the co-expression of other genes implicated in genetic, non-teratogenic, limb reduction defects (LRD), such as *Tbx5*, *Esco2*, *Recql4*, and *Sall4*; *Crbn* and *Sall4* were shown to have a moderate co-expression correlation, which is affected after thalidomide exposure. Hence, even though the classic TE phenotype is not identified in mice, a deregulatory Crbn-induced mechanism is suggested in this animal. Functional studies are necessary, especially evaluating the genes responsible for LRD syndromes and their interaction with thalidomide–Cereblon.

## Introduction

Since the 1960s, when thalidomide teratogenicity was discovered, many attempts have been tried to identify its molecular mechanisms, aiming to use this knowledge to avoid new cases of the embryopathy. However, fully understanding how thalidomide causes malformations remains an urgent challenge (Asatsuma-Okumura et al., 2019).

One of the many aspects that intrigue scientists is the variability of thalidomide teratogenesis among species (Table 1). The most commonly used animal models, rat and mouse, do not develop the classic thalidomide embryopathy (TE) phenotype, identified in a range of organisms including zebrafish (Siamwala et al., 2012), chicken (Therapontos et al., 2009), frogs (Fort et al., 2000), rabbits (Fabro et al., 1967), sea urchins (Reichard-Brown et al., 2009), and opossum (Sorensen et al., 2017). Mice were tested even in a dose of 4,000 mg/kg and did not develop TE, whereas humans are sensitive to its teratogenesis in a dose of 0.5 mg/kg (Cahen, 1966; Stephens and Fillmore, 2000). Other insensitive species are bushbabies, pigs, and cats (Fink et al., 2018). However, the original studies with bushbabies and pigs have a small sample (*n* = 4) (Jonsson, 1972; Butler, 1977), and experiments with pregnant cats treated with thalidomide resulted in fetal loss and a variety of cardiovascular anomalies in the offspring (Khera, 1975).

**TABLE 1:** Comparative table of the effects of thalidomide, and its analogs, exposure in different animal models.

Animal model	Thalidomide teratogenesis	Thalidomide analogs teratogenesis	References
**Zebrafish** (*Danio rerio*)	Thalidomide is teratogenic, causing fin anomalies, and delayed development	CPS49 and lenalidomide are teratogenic, pomalidomide is non-teratogenic.	Siamwala et al., 2012; Mahony et al., 2013
**Frog** (*Xenopus laevis*)	Observation of malformations, including abnormal limb bud	NA	Fort et al., 2000
**Chicken** (*Gallus gallus*)	Observation of limb reduction defects	CPS49 causes wing defects such as truncations and phocomelia. Lenalidomide is teratogenic and pomalidomide is non-teratogenic	Stephens, 2009; Therapontos et al., 2009; Mahony et al., 2013
**Opossum** (*Monodelphis domestica*)	Observation of limb anomalies modeling human Thalidomide Embryopathy	NA	Sorensen et al., 2017
**Bushbaby** (*Otolemur crassicaudatus*)	Embryos exposed to 20 mg/kg of thalidomide between days 16 and 42 of pregnancy do not present teratogenic response	NA	Butler, 1977
**Armadillo** (*Dasypus novemcinctus*)	Thalidomide induces phocomelia and amelia in *Dasypus novemcinctus* (north-American armadillo)	NA	Marin-Padilla and Benirschke, 1963
**Sheep** (*Ovis aries*)	Thalidomide is teratogenic in sheep	NA	Ruckebusch, 1983
**Rabbit** (*Oryctolagus cuniculus*)	Thalidomide induces limb anomalies and embryo lethality in in Chinchilla and New Zealand white rabbits.	Lenalidomide caused embryo lethality, but no anomalies. Pomalidomide caused limb and cardiac anomalies in rabbits.	Somers, 1962; Fabro et al., 1967; Christian et al., 2007; Celgene, 2013,2015.
**Hamster** (*Mesocricetus auratus*)	Thalidomide is teratogenic in certain inbred strains of hamsters, including *Mesocricetus auratus*	NA	Homburger et al., 1965
**Rat** (*Rattus norvegicus*)	Thalidomide does not induce limb anomalies in rats.	Lenalidomide had no effect in rat. Pomalidomide is teratogenic, causing skeletal, thyroid and urinary bladder malformations.	Bauer et al., 1998; Celgene, 2013,2015
**Mouse** (*Mus musculus*)	Thalidomide is non-teratogenic in mice	NA	Cahen, 1966; Stephens et al., 2000
**Monkey** (*Macaca mulatta, Macaca fascicularis, Callithrix jacchus*)	Thalidomide causes limb anomalies in marmoset, crab-eating, and Rhesus monkeys, similar to the ones observed in humans after thalidomide exposure	Lenalidomide caused limb anomalies alike the anomalies observed in humans after thalidomide exposure.	Delahunt and Lassen, 1964; Merker et al., 1988; Ema et al., 2010; Celgene, 2013

*NA, Not Available.*

Molecular mechanisms previously identified in thalidomide are different across species, such as the induction of oxidative stress (Hansen and Harris, 2004), driven by several genes including *NFKB1*, *NFKB2*, *DKK1*, *GSTP1*, and *GSS*. Other mechanisms for thalidomide teratogenesis have been proposed, such as anti-angiogenesis, which led to several studies evaluating the role of the vascular endothelium mainly expressing genes (*KDR*, *VEGFA*, and *CDH5*) and nitric oxide synthase genes *NOS2* and *NOS3* (Therapontos et al., 2009; Siamwala et al., 2012). All the studies have aimed to understand how the molecular disruptions directed by thalidomide could impact in limb development (Hansen and Harris, 2004; Therapontos et al., 2009; Ito et al., 2010; Siamwala et al., 2012). However, only recently, Donovan et al. (2018) and Matyskiela et al. (2018) elucidated an important mechanism that has helped in the understanding of this mystery: Cereblon, a protein which is a thalidomide primary target (Ito et al., 2010), mediates SALL4 degradation (Donovan et al., 2018; Matyskiela et al., 2018).

SALL4 is a transcription factor (TF) involved in limb development; in humans, loss-of-function mutations in *SALL4* cause Duane-radial ray syndrome (Kohlhase et al., 2002), an autosomal recessive condition characterized by limb anomalies, similar to the ones observed in TE (Kohlhase et al., 2003). Because of the pattern of anomalies, TE is considered a phenocopy of other genetic syndromes, mainly characterized by limb reduction defects (Cassina et al., 2017), caused by mutations in *ESCO2*, *TBX5*, *RBM8A*, and *RECQL4* genes (Li et al., 1997; Vega et al., 2005; Van Maldergem et al., 2006).

Recently, it was proposed that, in mice, variants in *Crbn* and *Sall4* genes interrupt the degradation of Sall4 protein mediated by Cereblon–thalidomide (Donovan et al., 2018; Matyskiela et al., 2018). In this scenario, genetic alterations in both *SALL4* and *CRBN* would be pivotal for thalidomide teratogenic activity (Gao et al., 2020). However, only a few species were evaluated in the mentioned research. Besides SALL4, other neosubstrates have recently been proposed, including limb development genes such as *MEIS2* (Fischer et al., 2014) and *TP63* (Asatsuma-Okumura et al., 2019).

Before it was identified as a thalidomide primary target (Ito et al., 2010), a Cereblon stop-codon mutation was implicated in a mild mental retardation autosomal recessive disorder (Higgins et al., 2004). Notwithstanding, its canonical function is related to a E3–ubiquitin–ligase complex, which comprises DDB1, ROC1, and CUL4A proteins as well; Cereblon acts as a substrate receptor, ubiquitinating proteins to be degraded (Angers et al., 2006). In mouse brains, Cereblon mRNA was detected especially in serotoninergic and adrenergic neurons, as well as in hippocampal and cerebellum regions (Aizawa et al., 2011). Worthy of note is that *Crbn*-null mice are viable and do not present major malformations (Rajadhyaksha et al., 2012; Lee et al., 2013); however, memory and learning deficits are apparent in these animals (Bavley et al., 2018). Because of Cereblon binding and the outstanding immunomodulatory capacity of thalidomide, the drug has been implicated in the treatment of neurodegenerative and neuropsychiatric disorders; however, thalidomide neuroimmunomodulatory mechanisms do not appear to be Cereblon-dependent and probably occur through TNFa mRNA degradation (Jung et al., 2021).

Notwithstanding, TE in humans is considered heterogeneous, because the phenotypic presentation is variable, an effect that is not explained only by *SALL4* or *CRBN* variants (Gomes et al., 2019; Kowalski et al., 2020). Considering TE intra- and interspecific variability, the aim of this research was to perform a comparative analysis in the main proteins already associated with thalidomide mechanisms of action, across different species, affected or not by classic TE. We have evaluated 42 genes, including *SALL4*, regarding synteny, neighborhood, and protein conservation, which might be relevant in the understanding of TE divergences. Moreover, we also performed differential gene expression and co-expression analyses, focused in the mice to better comprehend the thalidomide action mechanisms in this species; we combined these bioinformatics analyses with systems biology strategies, to evaluate the main targets of thalidomide exposure in mice and rats.

## Materials and Methods

**FIGURE 1:**
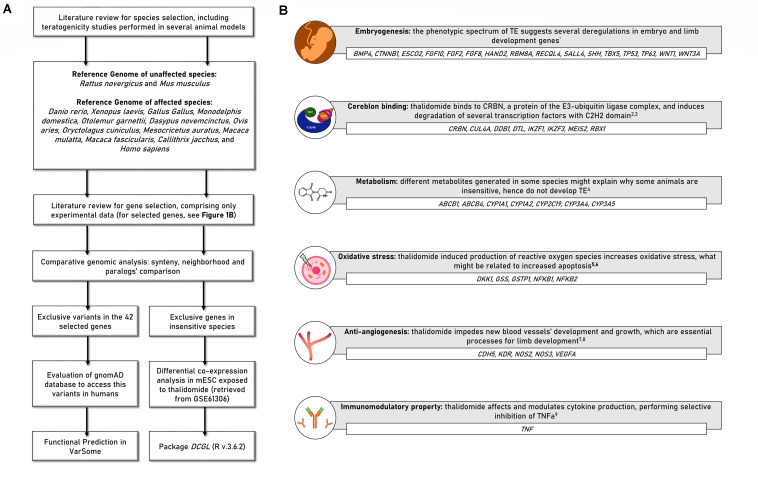
**(A)** Scheme presenting the study delineation and analyses. **(B)** Forty-two candidate genes selected for the comparative analyses. *BMP4*, Bone morphogenetic protein 4; *CTNNB1*, Catenin beta-1; *ESCO2*, Establishment of Sister Chromatid Cohesion N-Acetyltransferase 2; *FGF10*, Fibroblast growth factor 10; *FGF2*, Fibroblast Growth Factor 2; *FGF8*, Fibroblast Growth Factor 8; *HAND2*, Heart And Neural Crest Derivatives Expressed 2; *RBM8A*, RNA Binding Motif Protein 8A; *RECQL4*, RecQ Like Helicase 4; *SALL4*, Sal-like protein 4; *SHH*, Sonic Hedgehog Signaling Molecule; *TBX5*, T-Box Transcription Factor 5; *TP53*, Tumor Protein P53; *TP63*, Tumor Protein P63; *WNT1*, Wnt Family Member 1; *WNT3A*, Wnt Family Member 3A; *CRBN*, Cereblon; *CUL4A*, Cullin 4A; *DDB1*, Damage Specific DNA Binding Protein 1; *DTL*, Denticleless E3 Ubiquitin Protein Ligase Homolog; *IKZF1*, IKAROS Family Zinc Finger 1; *IKZF3*, IKAROS Family Zinc Finger 3; *MEIS2*, Meis Homeobox 2; *RBX1*, Ring-Box 1; *ABCB1*, ATP Binding Cassette Subfamily B Member 1; *ABCB4*, ATP Binding Cassette Subfamily B Member 4; *CYP1A1*, Cytochrome P450 1A1; *CYP1A2*, Cytochrome P450 1A2; *CYP2C19*, Cytochrome P450 2C19; *CYP3A4*, Cytochrome P450 3A4; *CYP3A5*, Cytochrome P450 3A5; *CDH5*, Cadherin 5; *KDR*, Kinase Insert Domain Receptor; *NOS2*, Nitric oxide synthase 2; *NOS3*, Nitric Oxide Synthase 3; *VEGFA*, Vascular endothelial growth factor A; *DKK1*, Dickkopf WNT Signaling Pathway Inhibitor 1; *GSS*, Glutathione synthetase; *GSTP1*, Glutathione S-transferase P; *NFKB1*, Nuclear factor NF-kappa-B p105 subunit; *NFKB2*, Nuclear factor NF-kappa-B p100 subunit; *TNF*, Tumor necrosis factor; TE, thalidomide embryopathy. References—^1^Knobloch et al., 2011; ^2^Ito et al., 2010; ^3^Donovan et al., 2018; ^4^Stephens et al., 2000; ^5^Hansen and Harris, 2004; ^6^Knobloch et al., 2007; ^7^D’Amato et al., 1994; ^8^Vargesson, 2009; ^9^Sampaio et al., 1991.

A full description of the performed analyses is available in Supplementary File 1. Figure 1A presents a step-by-step scheme of the analysis.

### Species and Gene Selection

Fourteen species were included according to previous reports regarding thalidomide exposure during embryo development (Table 1). Forty-two candidate genes were selected from previous studies in animal models; hence, only genes previously accessed by *in vitro* or *in vivo* experiments were included (D’Amato et al., 1994; Hansen and Harris, 2004; Therapontos et al., 2009; Ito et al., 2010; Siamwala et al., 2012; Donovan et al., 2018; Matyskiela et al., 2018). These genes are related to (1) thalidomide metabolism; (2) embryonic and/or limb development; and (3) thalidomide molecular mechanisms: anti-angiogenesis, oxidative stress, binding to Cereblon protein, and immunomodulatory property (Figure 1B).

### Comparative Genomics Analysis

**FIGURE 2:**
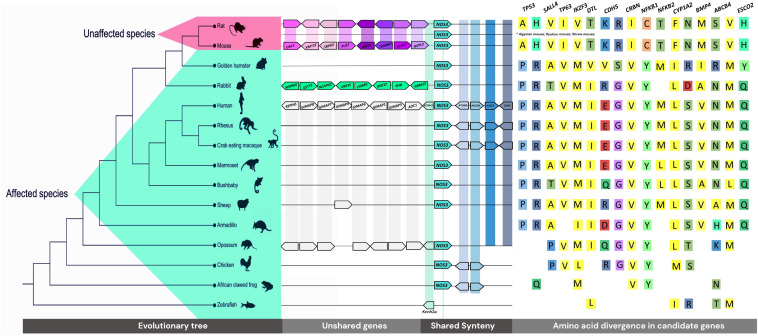
Genomic tree of the evaluated species, synteny and neighborhood of *NOS3* gene, and the main variants encountered in mice and rats, which are reported in gnomAD database.

Synteny, neighborhood reports, and comparison of paralog amount were done for all 42 genes based on genomic databases. The main comparative method used was searching for differences present in the genetic maps and sequences of 12 affected vs. two non-affected species (Table 1 and Figure 2). Thus, species-specific variations were not described. We retrieved transcript sequences from Ensembl release 100 and/or BLAST on NCBI. We performed alignments using MUSCLE (Edgar, 2004) in MEGA 7 (Kumar et al., 2016). gnomAD was assessed to verify variants in humans. Functional prediction was performed in VarSome. PubTator, Ensembl, and PharmGKB databases were used to evaluate clinical and/or pharmacogenetic associations.

### Differential Co-expression Analysis

Differential co-expression analysis was performed in the GSE61306 study (Gao et al., 2015), obtained from the GEO database. RNA from mESC was collected 24, 48, and 72 h after thalidomide or saline exposure. Confirmatory analyses were performed in valproic acid, retinoic acid, or warfarin-exposed mESC, available in the ArrayExpress database (accession numbers: E-TABM-903, E-TABM-1205, and E-MTAB-300). *DCGL* R package was used for differential co-expression analysis (Yang et al., 2013). Gene–gene co-expression was evaluated using Pearson correlation, including only Pearson’s r of at least |± 0.5| in exposed or control samples (Mukaka, 2012). *P*-values of < 0.05 were included, after false discovery rate (FDR) adjustment. For the TF analysis, we incorporated the TRRUST database (Han et al., 2015) in the *DCGL* package, to obtain *Mus musculus* interactions. Co-expression networks were assembled Cytoscape v.3.7.2. Heatmaps were generated using *diffcoexp* and *ggplot2* R packages.

## Results

### Synteny, Neighborhood, and Gene Copy Number Differences Across Species

Twelve species who develop TE and two non-affected (mouse and rat) were compared as two groups, affected vs. unaffected. Except for gene copy number differences, the results are related to differences of these groups. Hence, our synteny and neighborhood analysis either was conserved for all species or did not show a pattern of conservation between the groups. To illustrate, the vicinity of *SHH* in mouse and rat only has five downstream shared genes, while 20 upstream genes are divergent between these rodents. Consequently, we could not find any reasonable relation to thalidomide teratogenesis in such cases.

In contrast, an outstanding difference between the upstream neighborhood of the NOS3 gene in sensitive vs. insensitive species was identified: at least 20 genes composing that region differ from other species, in the same region (Figure 2). The rodents, except the golden hamster, share a subset of genes (Supplementary Table 1). The rabbit, exclusively, has the following genes related to the TE phenotype at that region: *WDR60, ESYT2, NCAPG2, UBE3C, LMBR1, RNF32, SHH, RBM33*, and *NOM1*. Other sensitive species share *GIMAPs* (GTPase, IMAP Family Members), which may be associated with Behcet’s syndrome, a condition treated with thalidomide (Shek and Lim, 2002), as well as cancer-related genes, such as *RARRES2* and *TMEM176A* (Supplementary Table 2).

We found a great difference between the number of copies of some genes related to metabolism among the animals, being affected or not by TE (Supplementary Table 3). *CYP2C19*, the main gene responsible for thalidomide metabolism (Teo et al., 2000), has 10 tandem copies (7 paralogs > 70% id) in the mouse, while only five (>76% id) in humans. *CYP3A4* and *CYP3A5* also vary in number of copies; mice have 9 (>68% id), while humans have 5 (>75% id) (Supplementary Table 4). There is a core set of 103 and 77 CYPs (Cytochrome P450’s-seed PF00067-pFam) in mouse and human genomes (Supplementary Table 5), respectively, enzymes involved directly in the oxidative metabolism.

### Gene Alignments Revealed Exclusive Unaffected Species’ Variants

Considering that thalidomide is not a selective pressure factor, positive selection sites were not searched. Thus, even a neutral substitution for intrinsic processes of the organisms could add some effect to molecular interactions of this medication into the cell. As a result, we found 299 amino acid substitutions exclusive in insensitive species in 34/42 analyzed genes. Proteins with a higher number of variants in the insensitive group were RECQL4 (65), SALL4 (35), CDH5 (22), KDR (18), and NOS2 (17). A detailed description of all substitutions and protein regions is available in Supplementary Table 6.

### Human Genetic Variability Shares Exclusive Unaffected Species’ Variants

For all the gene alignments, the reference genome sequence of each species was used. However, humans have great genetic variability; hence, missense alterations are known to exist in the human species, without being contemplated in the reference genome sequence. Based on this reasoning, all the variants exclusive for insensitive species, according to the comparative sequence alignment, were evaluated in the gnomAD database, aiming to identify similar alterations in humans.

Twenty-four variants, which led to the same missense alterations observed in mice and rats, were encountered in gnomAD registers, distributed across 13 genes (Supplementary Table 7). One missense substitution, p.P72A in *Trp53* of mice and rats, has been registered to occur after two possible genomic alterations in humans: (I) rs587782769, changing the first nucleotide of the codon; and (II) rs 1042522, modifying the second nucleotide of the codon. In mice and rats, the variant occurs by the exchange of the first nucleotide and the last nucleotide of the codon, being CCC in humans and GCA in mice and rats.

Functional prediction was performed in the VarSome database according to the criteria established by the American College of Medical Genetics (Richards et al., 2015). Fourteen variants were predicted as “likely benign” and 10 as “variants of unknown significance.” The variant rs587782769 of *TP53* was the only one predicted as “likely pathogenic,” because of previous associations with Li-Fraumeni syndrome, a predisposing syndrome of hereditary cancer. The other *TP53* variant that leads to *p*.P72A, rs 1042522, is of unknown significance in Li-Fraumeni syndrome. However, rs1042522 was the only variant identified with high frequency in human populations, having a global minor allele frequency of 0.6629. This was also the only alteration to be registered in the PharmGKB database, with a pharmacogenetic association for different drugs, all antineoplastic agents. The *CRBN* gene presented one variant of unknown significance, and *SALL4* presented two likely benign variants.

Overall, some variants encountered in insensitive species are reported as exclusive for these animals; however, when evaluating the immense variability of the human genome, it was possible to identify the same alterations in humans.

### *Crbn* Identified as a Deregulator of Gene-Pair Co-expression

**FIGURE 3:**
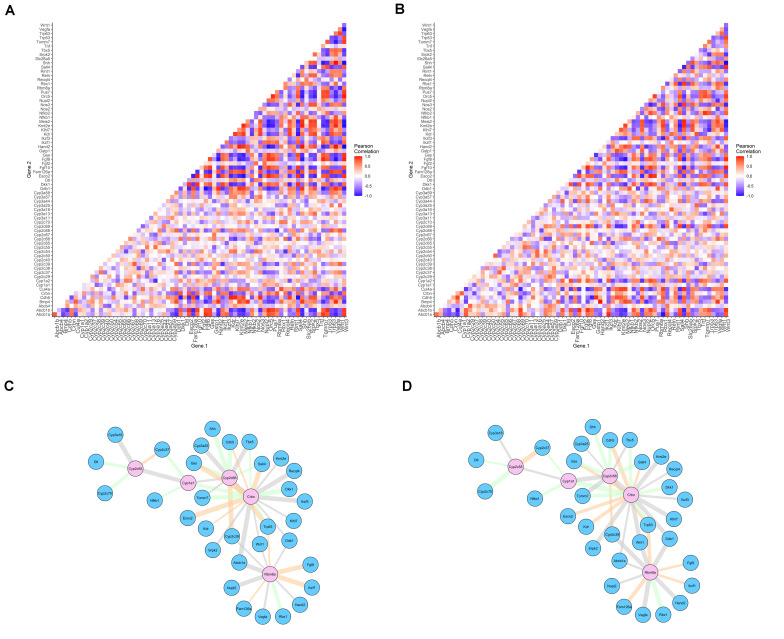
Pearson correlation for differentially co-expressed gene pairs in controls **(A)** and thalidomide **(B)** exposed cells. Networks of differentially co-expressed gene pairs for controls **(C)** and thalidomide **(D)** exposed cells. Legend: **(A,B)** positive correlations represented in red; negative correlations in blue; absence of correlation in white. **(C,D)** genes that drive co-expression are represented in pink nodes; the other gene that composes the pair is represented in blue nodes. Edges’ thickness represents Pearson’s *r* correlation coefficient; gray edges = switched opposites (*r* change > 1); orange edges = differentially signed (*r* change < 1, but altering correlation from positive to negative, or contrariwise); green edges = same signed (correlation was changed, but maintained as positive or negative).

The original results for the differential gene expression analysis are available in Gao et al. (2015). Here, we evaluated the co-expression of gene pairs through Pearson correlation, aiming to identify whether this correlation is disrupted in thalidomide exposure. Forty-two genes previously selected, and the genes identified to be exclusive of the insensitive species in the evolutionary analysis, were included, totalizing 71 genes. A heatmap comprising the Pearson correlation for all the gene pairs obtained is available in Figure 3A (for controls) and Figure 3B (for thalidomide exposure). Evaluating the red and blue areas, it is possible to visualize stronger expression correlations in controls, when compared to the white areas of the thalidomide-exposed sample heatmap. Nevertheless, aiming for a more robust statistical evaluation, a differential co-expression analysis was performed. This strategy aims to identify patterns of deregulation between genes, which could point to a dysfunctional pathway or mechanism and prioritize the main candidates in a list of hypothesized genes (de la Fuente, 2010; Savino et al., 2020).

Control vs. thalidomide-exposed mESC were evaluated, to determine the gene pairs with differential co-expression, and the gene of the pair that is probably driving this differential co-expression. As a result, 49 gene pairs differentially co-expressed were significant. The network of differential co-expression correlations can be visualized in Figure 3C (controls) and Figure 3D (thalidomide-exposed). Only *Crbn*, *Cyp2c68*, and *Rbm8a* genes were considered as significant drivers of the co-expression after FDR *P*-value adjustment (Supplementary Table 8). In 19 gene pairs, *Crbn* acted as the driver of this co-expression correlation; the other genes in the pairs are associated with limb reduction defects’ syndromes.

In regard to *Crbn-Sall4* Pearson correlation, it is set in *r* = 0.75 in control samples and *r* = –0.48, after thalidomide exposure. Hence, a positive correlation is altered to negative, with a correlation difference of 1.23. The biggest correlation difference encountered when *Crbn* was a co-expression gene driver was 1.37, for the *Crbn-Recql4* pair.

To better comprehend the co-expression correlations obtained, the same thalidomide-affected genes were analyzed in mESC cells exposed to three different teratogens, separately: valproic acid, retinoic acid, or warfarin. No statistically significant association was identified for evaluated genes (Supplementary Table 9). Another differential co-expression analysis was performed in the thalidomide-exposed mESC array (GSE61306), using a different set of genes, from the NF-kB pathway, known to be affected by thalidomide (Hansen and Harris, 2004). Five genes were identified as co-expression drivers, providing 70 gene pairs differentially co-expressed in control vs. thalidomide-exposure samples (Supplementary Table 10). These results indicate that the gene selection performed was careful and relevant to evaluate thalidomide-induced mechanisms; it also suggests that the positive association between *Crbn* and the limb reduction defects’ genes is not spurious.

In sequence, we evaluated which TFs could be regulators of the differentially co-expressed gene pairs. The results of this strategy are available in Supplementary Figure 1. *Ikzf1*, a C2H2 transcription factor degraded by thalidomide, is proposed as a driver of the co-expression, forming gene pairs with *Dkk1*, *Vegfa*, and *Kdr*. These genes have three other TFs in common: Bmi1, Ets1, and Runx2 (Supplementary Table 11), hence being suggested as potential regulators that may also alter the candidate gene expression.

In summary, differential co-expression analysis provided insights regarding thalidomide effects in mESC. First, *Crbn* drives the co-expression of other genes implicated in genetic, non-teratogenic, limb reduction defects, such as *Tbx5*, *Esco2*, *Recql4*, and *Sall4*; a fifth gene implicated in limb reduction defects, *Rbm8a*, was also considered a driver of the co-expression correlation. Second, *Crbn* and *Sall4* have a moderate co-expression correlation, which is affected in thalidomide exposure, even though the typical TE phenotype is not identified in mice. Finally, *Ikzf1* might be a regulator of two essential genes for angiogenesis: *Vegfa* and its receptor *Kdr.*

## Discussion

A comparative genomics analysis was associated with an evaluation of gene–gene co-expression to identify candidate genes for thalidomide teratogenesis variability across species. Twenty genes upstream *NOS3* were different when comparing sensitive and insensitive species neighborhoods. Co-expression analysis suggests that *Crbn* drives the co-expression of limb reduction defects’ genes, including Sall4, in mESC exposed to thalidomide. This might indicate a deregulation between Crbn-Sall4 even in a species known to be insensitive to thalidomide exposure.

During more than 50 years of research, properties of thalidomide and its analogs have been elucidated through animal model studies. Some mechanisms such as anti-angiogenesis (D’Amato et al., 1994; Therapontos et al., 2009) and oxidative stress (Hansen and Harris, 2004), evaluated in these studies, have an extremely relevant role in the whole understanding of the TE specificity. Mammals have an additional layer of complexity: the maternal—fetal interface, the mother metabolism genes affecting the drug availability and placental transfers. Recently, a new light was brought to the understanding of the TE scenario, due to the discovery of SALL4 degradation mediated by Cereblon–thalidomide (Donovan et al., 2018; Matyskiela et al., 2018). Mouse resistance to the TE development was justified by the presence of missense variants both in *CRBN* and in *SALL4* (Donovan et al., 2018; Matyskiela et al., 2018). In the present study, we identified differences among sensitive and insensitive animals in all mechanisms studied associated with thalidomide teratogenic effects, which could explain the heterogeneous outcome in the embryos, as we reported in humans (Kowalski et al., 2020).

Anti-angiogenesis is one of the most studied properties in TE. Our analysis of differential regulation suggests a co-expression correlation between an angiogenesis gene, *Vegfa*, and its receptor, *Kdr*, driven by the *Ikzf1* gene. Ikaros, the protein encoded by *Ikzf1*, degradation is demonstrated to be an important event in thalidomide therapeutics in multiple myeloma (Krönke et al., 2014). Furthermore, thalidomide anti-angiogenic property is suggested as independent of Cereblon binding (Beedie et al., 2020; Peach et al., 2020). Hence, the differential co-expression correlations and variants encountered in insensitive species might help to understand thalidomide anti-angiogenic effects in these animals.

Differences in anti-angiogenesis genes also have a relevant role in the whole understanding of TE specificity. In previous studies, evaluating individuals with TE, our group identified variants of susceptibility in *NOS3* (Vianna et al., 2013; Kowalski et al., 2016). Our synteny and neighborhood analyses demonstrated nearby genes to *NOS3* differ in unaffected rodent species, rabbit species, and other TE species. Eight genes close to *NOS3* in the rabbit are involved in conditions similar to TE, including acheiropody and polydactyly. A hypothesis is that alterations in the pattern of expression of these genes during development could favor the establishment of TE characteristics, considering TE conserved regulatory mechanisms. Interestingly, van den Boogaard et al. (2019) identified, in mice, a transcribed distal enhancer involved in the *KCNH2* gene regulation (the first gene upstream to *NOS3*, present in all species in Figure 2). *In vitro* knockdown of the ncRNA enhancer transcript results in reduced expression of *Kcnh2b* and two neighboring mRNAs, *Nos3* and *Abcb8*. Also, multiple partially redundantly active enhancers interfere in the complex regulatory landscape of *Kcnh2*. However, this evidence of a connection between the regulation and expression of *NOS3* immediately nearby genes refers to the syntenic region described in our results. Whether TE-tested species’ variable *NOS3* upstream regions share similar regulatory relationships according to their genes content remains to be explored.

Historical reports estimated that 20–50% of the human embryos exposed to thalidomide actually developed TE (Newman, 1986). Our studies have reported variants in the TE individuals when compared to Brazilians without congenital anomalies (Vianna et al., 2016; Gomes et al., 2019; Kowalski et al., 2020). The rare variants we identified in the canonical sequences of insensitive species were absent in that TE sample. The polymorphism rs1042522 of *TP53* was genotyped in TE and unaffected individuals; however, its frequency was similar in both groups (Gomes et al., 2017). Despite that, rs1042522 is a functionally well-characterized polymorphism (Dumont et al., 2003) that could also help to explain interspecific variability to thalidomide-teratogenesis. p53 is implicated as a regulator of the embryo’s susceptibility to teratogens (Torchinsky and Toder, 2010). Here, we demonstrate three variants in *CRBN* and 35 alterations in *SALL4* which are exclusive in mice and rats. The implication of these variants in Cereblon–thalidomide-induced degradation must be further evaluated.

The gene-specific approach used here to evaluate potential regulators in the co-expression analysis is the most fine-grained method in this type of analysis, usable for identification of genes that more strongly change their connectivity with other genes (Savino et al., 2020). In cancers, this approach has been used to prioritize therapeutic targets (Savino et al., 2020). Global analysis could be useful for the selection of new gene candidates; however, the prioritization of candidate regulators by biological relevance improves the chance of identifying true associated factors (Yang et al., 2013). Here, this prioritization was provided by accessing only genes that had been previously, experimentally validated in thalidomide exposure. Hence, differential co-expression analysis proposed *Crbn* as a gene that drives co-expression when evaluating different gene pairs.

In humans, *ESCO2*, *TBX5*, and *RECQL4* cause Roberts, Holt–Oram, and Baller–Gerold syndromes, respectively (Li et al., 1997; Vega et al., 2005; Van Maldergem et al., 2006). Besides, mutations in *Rbm8a* lead to thrombocytopenia-absent radius syndrome (Albers et al., 2012). All these genetic conditions are phenotypically very similar to TE (Cassina et al., 2017) and require differential diagnosis (Mansour et al., 2019). Despite being an exploratory result, this is the first suggestion of a thalidomide-driven effect in these genes, which might help to explain the phenotype similarities. It is known that *Sall4* expression is not reduced in thalidomide exposure, because the degrading mechanism occurs posttranslationally (Donovan et al., 2018). Here we demonstrate *Crbn-Sall4* gene co-expression being affected after thalidomide exposure in mESC. This result does not invalidate the idea of a genetic resistance driven by both *Crbn* and *Sall4* variants; however, the altered co-expression in mice raises the hypothesis whether this differential co-expression is also present in TE-affected species. A positive result in the affected species would point to an associated pre-transcriptional mechanism that may help to understand the TE susceptibility, as we have reported in humans (Kowalski et al., 2016).

Concluding, we found several differences between sensitive and insensitive animals regarding known mechanisms of thalidomide teratogenicity: we identified gene copy number divergences among species; non-syntenic genes at the upstream vicinity of *NOS3* in rodent, rabbit, and other TE species; and 299 variants in mice and rats within genes of interest. Some variants are in domains, and, until now, they are original data toward the thalidomide teratogenesis molecular context. Consequently, we could not predict if they would be neutral or functional for TE differential outcomes. However, 24 substitutions are rare missense alterations in humans. These results can inspire *in vitro* and *in vivo* additional investigations. We also performed a differential co-expression analysis in mESC exposed to thalidomide and identified *Crbn* as a gene that drives the expression correlation of a series of genes reported previously as affected by thalidomide, as well as genes related to limb reduction defects (of which TE is a phenocopy). These correlated gene pairs include *Crbn-Sall4*. Finally, differential regulation analysis suggests *Ikzf1* as a driver gene for co-expression as well, a result that deserves to be explored in depth in other studies, especially *in vivo*.

The research here conducted complements the recent results regarding thalidomide comparative teratogenesis. Our study is limited by the lack of experimental data of thalidomide exposure in other affected and unaffected species. However, this is an exploratory analysis that provides new insights for this challenge. The outburst of babies born with TE led to many changes in legislation concerning animal model research and drug commercialization around the world (Daemmrich, 2002). In order to guarantee safe commercial liberation of a medicine, all drugs must be tested at least in two animal species, one non-rodent (Cook and Fairweather, 1968), and results in these models must not be completely extrapolated to human species, once teratogenesis is not a species-specific process (De Santis et al., 2004). Hence, studies of comparative teratogenesis like this are important to trace strategies for avoiding a new thalidomide tragedy.

## Data Availability Statement

The original contributions presented in the study are included in the article/Supplementary Material, further inquiries can be directed to the corresponding author/s. Data studied was obtained from the public genomic databases Ensembl and GenBank (NCBI). Gene expression data is available in Gene Expression Omnibus (GEO) database, series number GSE61306. Confirmatory analyses were performed in valproic acid, retinoic acid or warfarin-exposed mESC, available in the ArrayExpress database (accession numbers: E-TABM-903, E-TABM-1205, and E-MTAB-300).

## Ethics Statement

Ethical review and approval was not required for the animal study because the present study uses only secondary data, available in GEO database (GSE61306) and ENA database (E-TABM-903, E-TABM-1205, and E-MTAB-300), as reported in the Data Availability Statement.

## Author Contributions

TK and GC-G contributed in devising the concept, designing and conducting the analyses, and writing the manuscript. JG contributed in devising the concept, designing the analysis, and performing the analyses. LS-F contributed in devising and supervising the analyses. LF, MR-M, VP-C, and FV contributed in devising the concept, designing the analyses, supervising the analyses, and correcting the manuscript. All authors discussed the results and contributed scientifically to the manuscript.

## Conflict of Interest

The authors declare that the research was conducted in the absence of any commercial or financial relationships that could be construed as a potential conflict of interest.

## References

[B1] Aizawa M. Abe Y. Ito T. Handa H. Nawa H. (2011). mRNA distribution of the thalidomide binding protein cereblon in adult mouse brain. *Neurosci. Res.* 69 343–347. 10.1016/j.neures.2010.12.019 21241746

[B2] Albers C. A. Paul D. S. Schulze H. Freson K. Stephens J. C. Smethurst P. A. (2012). Compound inheritance of a low-frequency regulatory SNP and a rare null mutation in exon-junction complex subunit RBM8A causes TAR syndrome. *Nat. Genet.* 44 s431–s432. 10.1038/ng.1083 22366785 PMC3428915

[B3] Angers S. Li T. Yi X. MacCoss M. J. Moon R. T. Zheng N. (2006). Molecular architecture and assembly of the DDB1-CUL4A ubiquitin ligase machinery. *Nature* 443 590–593. 10.1038/nature05175 16964240

[B4] Asatsuma-Okumura T. Ando H. De Simone M. Yamamoto J. Sato T. Shimizu N. (2019). p63 is a cereblon substrate involved in thalidomide teratogenicity. *Nat. Chem. Biol.* 15 1077–1084. 10.1038/s41589-019-0366-7 31591562

[B5] Bauer K. S. Dixon S. C. Figg W. D. (1998). Inhibition of angiogenesis by thalidomide requires metabolic activation, which is species-dependent. *Biochem. Pharmacol.* 55, 1827–1834. 10.1016/s0006-2952(98)00046-x 9714301

[B6] Bavley C. C. Rice R. C. Fischer D. K. Fakira A. K. Byrne M. Kosovsky M. (2018). Rescue of learning and memory deficits in the human nonsyndromic intellectual disability cereblon knock-out mouse model by targeting the AMP-activated protein kinase-mTORC1 translational pathway. *J. Neurosci.* 38 2780–2795. 10.1523/JNEUROSCI.0599-17.2018 29459374 PMC5852658

[B7] Beedie S. L. Huang P. A. Harris E. M. Strope J. D. Mahony C. Chau C. H. (2020). Role of cereblon in angiogenesis and in mediating the antiangiogenic activity of immunomodulatory drugs. *FASEB J.* 34 11395–11404. 10.1096/fj.201903060RR 32677118 PMC8819519

[B8] Butler H. (1977). The effect of thalidomide on a prosimian: the greater galago (Galago crassicaudatus). *J. Med. Primatol.* 6 319–324. 10.1159/000459764 609089

[B9] Cahen R. L. (1966). Experimental and clinical chemoteratogenesis. *Adv. Pharmacol.* 4 263–349. 10.1016/s1054-3589(08)60101-5 5333770

[B10] Cassina M. Cagnoli G. A. Zuccarello D. Di Gianantonio E. Clementi M. (2017). Human teratogens and genetic phenocopies. Understanding pathogenesis through human genes mutation. *Eur. J. Med. Genet.* 60 22–31. 10.1016/j.ejmg.2016.09.011 27639441

[B11] Celgene (2013). “Revlimid (lenalidomide) prescribing information,” in *Food and Drug Administration Access Data.* Available online at: https://www.accessdata.fda.gov/drugsatfda_docs/label/2012/021880s028lbl.pdf

[B12] Celgene (2015). “POMALYST (pomalidomide) prescribing information,” in *Food and Drug Administration Access Data*. Available online at: https://www.accessdata.fda.gov/drugsatfda_docs/label/2017/204026s019lbl.pdf

[B13] Christian M. S. Laskin O. L. Sharper V. Hoberman A. Stirling D. I. Latriano L. (2007). Evaluation of the developmental toxicity of lenalidomide in rabbits. *Birth Defects Res. B Dev. Reprod. Toxicol.* 80, 188–207. 10.1002/bdrb.20115 17570132

[B14] Cook M. J. Fairweather F. A. (1968). Methods used in teratogenic testing. *Lab. Anim.* 2 219–228. 10.1258/002367768781082834

[B15] Daemmrich A. (2002). A tale of two experts: thalidomide and political engagement in the United States and West Germany. *Soc. Hist. Med.* 15 137–158. 10.1093/shm/15.1.137 12625358

[B16] D’Amato R. J. Loughnan M. S. Flynn E. Folkman J. (1994). Thalidomide is an inhibitor of angiogenesis. *Proc. Natl. Acad. Sci. U. S. A.* 91 4082–4085. 10.1073/pnas.91.9.4082 7513432 PMC43727

[B17] de la Fuente A. (2010). From ‘differential expression’ to ‘differential networking’ – identification of dysfunctional regulatory networks in diseases. *Trends Genet.* 26 326–333. 10.1016/j.tig.2010.05.001 20570387

[B18] Delahunt C. S. Lassen L. J. (1964). Thalidomide syndrome in monkeys. *Science* 146, 1300–1305. 10.1126/science.146.3649.1300 14207455

[B19] De Santis M. Straface G. Carducci B. Cavaliere A. F. De Santis L. Lucchese A. (2004). Risk of drug-induced congenital defects. *Eur. J. Obstet. Gynecol. Reprod. Biol.* 117 10–19. 10.1016/j.ejogrb.2004.04.022 15474237

[B20] Donovan K. A. An J. Nowak R. P. Yuan J. C. Fink E. C. Berry B. C. (2018). Thalidomide promotes degradation of SALL4, a transcription factor implicated in Duane Radial Ray syndrome. *Elife* 7:e38430. 10.7554/eLife.38430 30067223 PMC6156078

[B21] Dumont P. Leu J. I. Della Pietra A. C. III George D. L. Murphy M. (2003). The codon 72 polymorphic variants of p53 have markedly different apoptotic potential. *Nat. Genet.* 33 357–365. 10.1038/ng1093 12567188

[B22] Edgar R. C. (2004). MUSCLE: multiple sequence alignment with high accuracy and high throughput. *Nucleic Acids Res.* 32 1792–1797. 10.1093/nar/gkh340 15034147 PMC390337

[B23] Ema M. Ise R. Kato H. Oneda S. Hirose A. Hirata-Koizumi M. (2010). Fetal malformations and early embryonic gene expression response in cynomolgus monkeys maternally exposed to thalidomide. *Reprod. Toxicol.* 29, 49–56. 10.1016/j.reprotox.2009.09.003 19751816

[B24] Fabro S. Smith R. L. Williams R. T. (1967). The fate of the hydrolysis products of thalidomide in the pregnant rabbit. *Biochem. J.* 104 570–574. 10.1042/bj1040570 6048799 PMC1270622

[B25] Fink E. C. McConkey M. Adams D. N. Haldar S. D. Kennedy J. A. Guirguis A. A. (2018). Crbn (I391V) is sufficient to confer in vivo sensitivity to thalidomide and its derivatives in mice. *Blood* 132 1535–1544. 10.1182/blood-2018-05-852798 30064974 PMC6172563

[B26] Fischer E. S. Böhm K. Lydeard J. R. Yang H. Stadler M. B. Cavadini S. (2014). Structure of the DDB1-CRBN E3 ubiquitin ligase in complex with thalidomide. *Nature* 512 49–53. 10.1038/nature13527 25043012 PMC4423819

[B27] Fort D. J. Stover E. L. Bantle J. A. Finch R. A. (2000). Evaluation of the developmental toxicity of thalidomide using frog embryo teratogenesis assay-xenopus (FETAX): biotransformation and detoxification. *Teratog. Carcinog. Mutagen.* 20 35–47. 10.1002/(sici)1520-6866200020:1<35::aid-tcm4<3.0.co;2-i 10607376

[B28] Gao S. Wang S. Fan R. Hu J. (2020). Recent advances in the molecular mechanism of thalidomide teratogenicity. *Biomed. Pharmacother.* 127:110114. 10.1016/j.biopha.2020.110114 32304852

[B29] Gao X. Sprando R. L. Yourick J. J. (2015). Transcriptomic changes in mouse embryonic stem cells exposed to thalidomide during spontaneous differentiation. *Data Brief* 4 199–202. 10.1016/j.dib.2015.05.014 26217789 PMC4510476

[B30] Gomes J. D. A. Kowalski T. W. Fraga L. R. Macedo G. S. Sanseverino M. T. V. Schuler-Faccini L. (2019). The role of ESCO2, SALL4 and TBX5 genes in the susceptibility to thalidomide teratogenesis. *Sci. Rep.* 9:11413. 10.1038/s41598-019-47739-8 31388035 PMC6684595

[B31] Gomes J. D. A. Kowalski T. W. Fraga L. R. Tovo-Rodrigues L. Sanseverino M. T. V. Schuler-Faccini L. (2017). Genetic susceptibility to thalidomide embryopathy in humans: study of candidate development genes. *Birth Defects Res.* 110 456–461. 10.1002/bdr2.1163 29193903

[B32] Han H. Shim H. Shin D. Shim J. E. Ko Y. Shin J. (2015). TRRUST: a reference database of human transcriptional regulatory interactions. *Sci. Rep.* 5:11432. 10.1038/srep11432 26066708 PMC4464350

[B33] Hansen J. M. Harris C. (2004). A novel hypothesis for thalidomide-induced limb teratogenesis: redox misregulation of the NF-kappaB pathway. *Antioxid. Redox Signal.* 6 1–14. 10.1089/152308604771978291 14713331

[B34] Higgins J. J. Pucilowska J. Lombardi R. Q. Rooney J. P. (2004). A mutation in a novel ATP-dependent Lon protease gene in a kindred with mild mental retardation. *Neurology* 63 1927–1931. 10.1212/01.wnl.0000146196.01316.a2 15557513 PMC1201536

[B35] Homburger F. Chaube S. Eppenberger M. Bogdonoff P. D. Nixon C. W. (1965). Susceptibility of certain inbred strains of hamsters to teratogenic effects of thalidomide. Toxicol. Appl. Pharmacol. 7, 686–693. 10.1016/0041-008x(65)90126-2 5866808

[B36] Ito T. Ando H. Suzuki T. Ogura T. Hotta K. Imamura Y. (2010). Identification of a primary target of thalidomide teratogenicity. *Science* 327 1345–1350. 10.1126/science.1177319 20223979

[B37] Jonsson B. G. (1972). Thalidomide teratology in swine: a preparatory study. *Acta Pharmacol. Toxicol. (Copenh.)* 31 24–26. 10.1111/j.1600-0773.1972.tb00692.x 5067387

[B38] Jung Y. J. Tweedie D. Scerba M. T. Kim D. S. Palmas M. F. Pisanu A. (2021). Repurposing immunomodulatory imide drugs (IMiDs) in neuropsychiatric and neurodegenerative disorders. *Front. Neurosci.* 15:656921. 10.3389/fnins.2021.656921 33854417 PMC8039148

[B39] Khera K. S. (1975). Fetal cardiovascular and other defects induced by thalidomide in cats. *Teratology* 11 65–69. 10.1002/tera.1420110108 124472

[B40] Knobloch J. Jungck D. Koch A. (2011). Apoptosis induction by thalidomide: critical for limb teratogenicity but therapeutic potential in idiopathic pulmonary fibrosis? *Curr. Mol. Pharmacol.* 4, 26–61. 10.2174/1874467211104010026 20958261

[B41] Knobloch J. Shaughnessy J. D. Jr. Rüther U. (2007). Thalidomide induces limb deformities by perturbing the Bmp/Dkk1/Wnt signaling pathway. *FASEB J.* 21, 1410–1421. 10.1096/fj.06-7603com 17283219

[B42] Kohlhase J. Heinrich M. Schubert L. Liebers M. Kispert A. Laccone F. (2002). Okihiro syndrome is caused by SALL4 mutations. *Hum. Mol. Genet.* 11 2979–2987. 10.1093/hmg/11.23.2979 12393809

[B43] Kohlhase J. Schubert L. Liebers M. Rauch A. Becker K. Mohammed S. N. (2003). Mutations at the SALL4 locus on chromosome 20 result in a range of clinically overlapping phenotypes, including Okihiro syndrome, Holt-Oram syndrome, acro-renal-ocular syndrome, and patients previously reported to represent thalidomide embryopathy. *J. Med. Genet.* 40 473–478. 10.1136/jmg.40.7.473 12843316 PMC1735528

[B44] Kowalski T. W. Fraga L. R. Tovo-Rodrigues L. Sanseverino M. T. Hutz M. H. Schuler-Faccini L. (2016). New findings in eNOS gene and Thalidomide Embryopathy suggest pre-transcriptional effect variants as susceptibility factors. *Sci. Rep.* 6:23404. 10.1038/srep23404 27004986 PMC4804217

[B45] Kowalski T. W. Gomes J. D. A. Garcia G. B. C. Fraga L. R. Paixao-Cortes V. R. Recamonde-Mendoza M. (2020). CRL4-cereblon complex in Thalidomide Embryopathy: a translational investigation. *Sci. Rep.* 10 851–851. 10.1038/s41598-020-57512-x 31964914 PMC6972723

[B46] Krönke J. Udeshi N. D. Narla A. Grauman P. Hurst S. N. McConkey M. (2014). Lenalidomide causes selective degradation of IKZF1 and IKZF3 in multiple myeloma cells. *Science* 343 301–305. 10.1126/science.1244851 24292625 PMC4077049

[B47] Kumar S. Stecher G. Tamura K. (2016). MEGA7: molecular evolutionary genetics analysis version 7.0 for bigger datasets. *Mol. Biol. Evol.* 33 1870–1874. 10.1093/molbev/msw054 27004904 PMC8210823

[B48] Lee K. M. Yang S. J. Kim Y. D. Choi Y. D. Nam J. H. Choi C. S. (2013). Disruption of the cereblon gene enhances hepatic AMPK activity and prevents high-fat diet-induced obesity and insulin resistance in mice. *Diabetes* 62 1855–1864. 10.2337/db12-1030 23349485 PMC3661653

[B49] Li Q. Y. Newbury-Ecob R. A. Terrett J. A. Wilson D. I. Curtis A. R. Yi C. H. (1997). Holt-Oram syndrome is caused by mutations in TBX5, a member of the Brachyury (T) gene family. *Nat. Genet.* 15 21–29. 10.1038/ng0197-21 8988164

[B50] Mahony C. Erskine L. Niven J. Greig N. H. Figg W. D. Vargesson N. (2013). Pomalidomide is nonteratogenic in chicken and zebrafish embryos and nonneurotoxic *in vitro. Proc. Nat. Acad. Sci. U.S.A.* 110, 12703–12708. 10.1073/pnas.1307684110 23858438 PMC3732931

[B51] Mansour S. Baple E. Hall C. M. (2019). A clinical review and introduction of the diagnostic algorithm for thalidomide embryopathy (DATE). *J. Hand. Surg. Eur. Vol.* 44 96–108. 10.1177/1753193418800631 30253685

[B52] Marin-Padilla M. Benirschke K. (1963). Thalidomide induced alterations in the blastocyst and placenta of the armadillo, dasypus novemcinctus mexicanus, including a choriocarcinoma. *Am. J. Pathol.* 43, 999–1016. 14099460 PMC1949780

[B53] Matyskiela M. E. Couto S. Zheng X. Lu G. Hui J. Stamp K. (2018). SALL4 mediates teratogenicity as a thalidomide-dependent cereblon substrate. *Nat. Chem. Biol.* 14 981–987. 10.1038/s41589-018-0129-x 30190590

[B54] Merker H. J. Heger W. Sames K. Stürje H. Neubert D. (1988). Embryotoxic effects of thalidomide-derivatives in the non-human primate Callithrix jacchus. I. Effects of 3-(1,3-dihydro-1-oxo-2H-isoindol-2-yl)-2,6-dioxopiperidine (EM12) on skeletal development. *Arch. Toxicol.* 61, 165–179. 10.1007/BF00316631 3355362

[B55] Mukaka M. M. (2012). Statistics corner: a guide to appropriate use of correlation coefficient in medical research. *Malawi Med. J.* 24 69–71. 23638278 PMC3576830

[B56] Newman C. G. (1986). The thalidomide syndrome: risks of exposure and spectrum of malformations. *Clin. Perinatol.* 13 555–573. 10.1016/s0095-5108(18)30810-8 3533365

[B57] Peach M. L. Beedie S. L. Chau C. H. Collins M. K. Markolovic S. Luo W. (2020). Antiangiogenic activity and in silico cereblon binding analysis of novel thalidomide analogs. *Molecules* 25:5683. 10.3390/molecules25235683 33276504 PMC7730988

[B58] Rajadhyaksha A. M. Ra S. Kishinevsky S. Lee A. S. Romanienko P. DuBoff M. (2012). Behavioral characterization of cereblon forebrain-specific conditional null mice: a model for human non-syndromic intellectual disability. *Behav. Brain Res.* 226 428–434. 10.1016/j.bbr.2011.09.039 21995942 PMC5864115

[B59] Reichard-Brown J. L. Spinner H. McBride K. (2009). Sea urchin embryos exposed to thalidomide during early cleavage exhibit abnormal morphogenesis later in development. *Birth Defects Res. B Dev. Reprod. Toxicol.* 86 496–505. 10.1002/bdrb.20215 20025048

[B60] Richards S. Aziz N. Bale S. Bick D. Das S. Gastier-Foster J. (2015). Standards and guidelines for the interpretation of sequence variants: a joint consensus recommendation of the American College of Medical Genetics and Genomics and the Association for Molecular Pathology. *Genet. Med.* 17 405–424. 10.1038/gim.2015.30 25741868 PMC4544753

[B61] Ruckebusch Y. (1983). Pharmacology of reticulo-ruminal motor function. *J. Vet. Pharmacol. Ther.* 6, 245–272. 10.1111/j.1365-2885.1983.tb00001.x 6142121

[B62] Sampaio E. P. Sarno E. N. Galilly R. Cohn Z. A. Kaplan G. (1991). Thalidomide selectively inhibits tumor necrosis factor alpha production by stimulated human monocytes. *J. Exp. Med.* 173, 699–703. 10.1084/jem.173.3.699 1997652 PMC2118820

[B63] Savino A. Provero P. Poli V. (2020). Differential Co-expression analyses allow the identification of critical signalling pathways altered during tumour transformation and progression. *Int. J. Mol. Sci.* 21:9461. 10.3390/ijms21249461 33322692 PMC7764314

[B64] Shek L. P. Lim D. L. (2002). Thalidomide in Behçet’s disease. *Biomed. Pharmacother.* 56 31–35. 10.1016/s0753-3322(01)00154-8 11908493

[B65] Siamwala J. H. Veeriah V. Priya M. K. Rajendran S. Saran U. Sinha S. (2012). Nitric oxide rescues thalidomide mediated teratogenicity. *Sci. Rep.* 2:679. 10.1038/srep00679 22997553 PMC3447183

[B66] Somers G. S. (1962). Thalidomide and congenital abnormalities. *Lancet* 1, 912–913. 10.1016/s0140-6736(62)91943-8 13915092

[B67] Sorensen D. Sackett A. Urban D. J. Maier J. Vargesson N. Sears K. E. (2017). A new mammalian model system for thalidomide teratogenesis: monodelphis domestica. *Reprod. Toxicol.* 70 126–132. 10.1016/j.reprotox.2017.01.010 28130151

[B68] Stephens T. D. (2009). The effect of thalidomide in chicken embryos. *Birth Defects Res. B Dev. Reprod. Toxicol.* 85, 725–731. 10.1002/bdra.20597 19645049

[B69] Stephens T. D. Bunde C. J. Fillmore B. J. (2000). Mechanism of action in thalidomide teratogenesis. *Biochem. Pharmacol.* 59, 1489–1499. 10.1016/s0006-2952(99)00388-3 10799645

[B70] Stephens T. D. Fillmore B. J. (2000). Hypothesis: thalidomide embryopathy-proposed mechanism of action. *Teratology* 61 189–195. 10.1002/(SICI)1096-9926(200003)61:3<189::AID-TERA6<3.0.CO;2-W 10661908

[B71] Teo S. K. Sabourin P. J. O’Brien K. Kook K. A. Thomas S. D. (2000). Metabolism of thalidomide in human microsomes, cloned human cytochrome P-450 isozymes, and Hansen’s disease patients. *J. Biochem. Mol. Toxicol.* 14 140–147. 10.1002/(sici)1099-0461200014:3<140::aid-jbt3<3.0.co;2-p 10711629

[B72] Therapontos C. Erskine L. Gardner E. R. Figg W. D. Vargesson N. (2009). Thalidomide induces limb defects by preventing angiogenic outgrowth during early limb formation. *Proc. Natl. Acad. Sci. U. S. A.* 106 8573–8578. 10.1073/pnas.0901505106 19433787 PMC2688998

[B73] Torchinsky A. Toder V. (2010). Mechanisms of the embryo’s response to embryopathic stressors: a focus on p53. *J. Reprod. Immunol.* 85 76–80. 10.1016/j.jri.2010.01.003 20227113

[B74] van den Boogaard M. van Weerd J. H. Bawazeer A. C. Hooijkaas I. B. van de Werken H. Tessadori F. (2019). Identification and characterization of a transcribed distal enhancer involved in cardiac Kcnh2 regulation. *Cell Rep.* 28 2704–2714.e5. 10.1016/j.celrep.2019.08.007 31484079

[B75] Van Maldergem L. Siitonen H. A. Jalkh N. Chouery E. De Roy M. Delague V. (2006). Revisiting the craniosynostosis-radial ray hypoplasia association: Baller-Gerold syndrome caused by mutations in the RECQL4 gene. *J. Med. Genet.* 43 148–152. 10.1136/jmg.2005.031781 15964893 PMC2564634

[B76] Vargesson N. (2009). Thalidomide-induced limb defects: resolving a 50-year-old puzzle. *Bioessays* 31, 1327–1336. 10.1002/bies.200900103 19921660

[B77] Vega H. Waisfisz Q. Gordillo M. Sakai N. Yanagihara I. Yamada M. (2005). Roberts syndrome is caused by mutations in ESCO2, a human homolog of yeast ECO1 that is essential for the establishment of sister chromatid cohesion. *Nat. Genet.* 37 468–470. 10.1038/ng1548 15821733

[B78] Vianna F. S. Fraga L. R. Tovo-Rodrigues L. Tagliani-Ribeiro A. Biondi F. Maximino C. M. (2013). Polymorphisms in the endothelial nitric oxide synthase gene in thalidomide embryopathy. *Nitric Oxide* 35 89–92. 10.1016/j.niox.2013.09.002 24055736

[B79] Vianna F. S. Kowalski T. W. Tovo-Rodrigues L. Tagliani-Ribeiro A. Godoy B. A. Fraga L. R. (2016). Genomic and in silico analyses of CRBN gene and thalidomide embryopathy in humans. *Reprod. Toxicol.* 66 99–106. 10.1016/j.reprotox.2016.10.003 27751757

[B80] Yang J. Yu H. Liu B. H. Zhao Z. Liu L. Ma L. X. (2013). DCGL v2.0: an R package for unveiling differential regulation from differential co-expression. *PLoS One* 8:e79729. 10.1371/journal.pone.0079729 24278165 PMC3835854

