# Supplementary material for: Comparative Genomics Identifies Putative Interspecies Mechanisms Underlying Crbn-Sall4-Linked Thalidomide Embryopathy
Source: Front Genet. 2021 Jun 23;12:680217. doi: 10.3389/fgene.2021.680217 (PMC8262662; doi:10.3389/fgene.2021.680217)
Supplement: Supplementary file 1 [file Image_1.pdf]

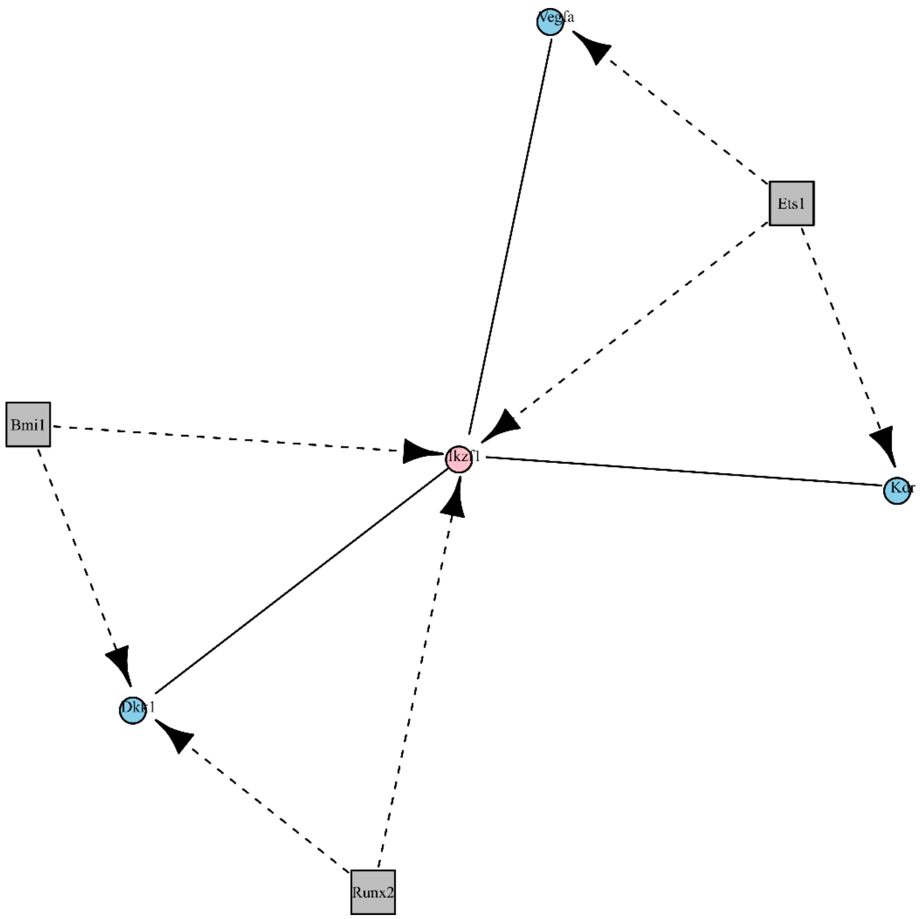

Supplemental Figure 1. Differential regulation analysis, presenting transcription factor *Ikzf1* (pink circle) as a gene driving the differential co-expression of *Dkk1*, *Kdr*, and *Vegfa* (blue circles), and other associated transcription factors (grey squares).
