# Supplementary material for: Comparative Genomics Identifies Putative Interspecies Mechanisms Underlying Crbn-Sall4-Linked Thalidomide Embryopathy
Source: Front Genet. 2021 Jun 23;12:680217. doi: 10.3389/fgene.2021.680217 (PMC8262662; doi:10.3389/fgene.2021.680217)
Supplement: Supplementary file 3 [file Data_Sheet_2.pdf]

Supplemental File 1

1.1 Species and Genes Selection

Genes and species were selected after an intense literature review in PubMed (National Institute of Health, NIH, USA). Fourteen species were selected according to previous reports on thalidomide exposure during embryo development (Table 1). Forty-two candidate genes were selected from previous studies in animal models, hence only genes previously accessed by *in vitro* or *in vivo* experiments were included (D'Amato et al., 1994; Hansen and Harris, 2004; Therapontos et al., 2009; Ito et al., 2010; Siamwala et al., 2012; Donovan et al., 2018; Matyskiela et al., 2018). These genes are related to (1) thalidomide metabolism; (2) embryonic and/or limb development; (3) thalidomide molecular mechanisms: anti-angiogenesis, oxidative stress, binding to Cereblon protein and immunomodulatory property (Figure 1B). The selected genes are available in Figure 1.

We included species according to availability of high-quality sequences obtained from the genomic databases (Table 1; Figure 2). Consequently, other previously tested species, such as the bonnet macaque (*Macaca radiata*), grivet (*Chlorocebus aethiops*), stump-tailed macaque (*Macaca arctoides*), Japanese macaque (*Macaca fuscata*), yellow baboon (*Papio cynocephalus*), brown greater galago (*Otolemur garnettii*), and Senegal bushbaby (*Galago senegalensis*) were not included in this study due to unavailability of transcript sequences. In addition, the African clawed frog (*Xenopus laevis*) is absent in some alignments for the same reason. Yet, pigs and cats, with controversial study designs (Jonsson, 1972; Khera, 1975), were excluded from the analysis. We provided sequences' identifiers and species for each candidate gene in details in Supplemental Table 7.

1.2 Comparative Genomics Analysis

Synteny, neighborhood reports, and comparison of paralogs amount were done for all forty-two candidate genes based on Ensembl v.93, Genomicus v.93, and UCSC Genome Browser. The main comparative method used was searching for differences present in the genetic maps and sequences of twelve affected *versus* two non-affected species (Table 1; Figure 2). Thus, species-specific variations were not described. For bushbaby, we highlight that thalidomide was tested in *Otolemur crassicaudatus*, however, this genome is not available for comparison, hence *Otolemur garnettii* was used (Figure 2).

We retrieved transcript sequences from Ensembl release 100 and/or through BLAST (Basic Local Alignment Search Tool) on NCBI (National Center for Biotechnology Information). We performed alignments using theMUSCLE algorithm (Edgar, 2004) in MEGA 7 (Molecular Evolutionary Genetics Analysis version 7) (Kumar et al., 2016). The searches for variants only found in unaffected species were done by visualization of the aligned protein sequences using MEGA 7 and Bioedit version 7.2.5 (Hall, 1999). The variants' positions were manually annotated using the human canonical sequence of each gene as reference. Also, we double-checked them on UniProt (uniprot.org), searching for human, mouse, and rat sequences. We described protein regions and domains where the reported variants are encountered based on UniProt and Pfam (pfam.xfam.org/) (Supplemental Table 7). Finally, gnomAD was assessed to verify whether a variant was described in humans, evaluating all populations available.

Functional prediction was performed with 12 predictors available in VarSome by inserting the rs ID for each variant encountered in gnomAD. PubTator, Ensembl, and PharmGKB databases were also used to evaluate clinical and/or pharmacogenetics associations; searched were conducted by inserting the rs ID for the variants encountered in gnomAD and all the citations available were accessed.

1.3 Expression Analysis

To evaluate gene expression, we searched for thalidomide-exposure studies available in Gene Expression Omnibus (GEO) database. As inclusion criteria, the thalidomide-exposed group should have a n>5, because of the co-expression analysis, which should not be processed in an assay with less than five samples per group (for more information, please see https://horvath.genetics.ucla.edu/html/CoexpressionNetwork/Rpackages/WGCNA/). As exclusion criteria, we did not consider any study accessing thalidomide in tumor-cells, because the aim of this study was to evaluate the effect in the embryonic cells. Hence, differential Gene Expression (DGE) and Differential Co-Expression (DCE) analyses were performed in the only study that met the criteria, available under dataset number GSE61306 (Gao et al., 2015), obtained from the GEO database. Confirmatory analyses were performed in microarrays evaluating valproic acid, retinoic acid or warfarin-exposed mESC, available in the ArrayExpress database (accession numbers: E-TABM-903, E-TABM-1205, and E-MTAB-300). RNA from mESC was collected 24, 48, and 72 hours after thalidomide or saline exposure. Differential gene expression analyses were executed in R v.3.6.2, using *oligo*, *affy* and *limma* packages, and the P-values obtained were adjusted for false discovery rate (FDR) to verify if any of the genes selected for the present study were differentially expressed. The result was negative, and all the positive associations are available in the original study from which the expression data was retrieved (Gao et al., 2015).

DCGL R package was used for differential co-expression analysis (Yang et al., 2013). *DCGL* provides consolidated algorithms to perform both global and gene-specific analyses (Savino et al., 2020); the latter was the one chosen for this study. Hence, gene-gene co-expression was evaluated using Pearson correlation, and only moderate, high, or super-high correlations were considered as biologically significant, hence including Pearson's r of at least $|\pm0.5|$ in exposed or control samples (Mukaka et al., 2012). To perform the analysis, samples were downloaded from GEO database into R. Gene symbols were added by accessing the platform library, also available in GEO database. The step-by-step analysis is available in the package vignette, provided as a supplementary file in Yang et al. (2013) package description. We used the default functions suggested by the package developers for all the analyses. With this method, it is possible to access gene-pairs that are co-differentially expressed, meaning the expression correlation between them was altered after the exposure to thalidomide; an alteration that was not identified in the control samples. The gene-pair correlated co-expression is presented as a DCL in the package script. In addition to these alterations, *DCGL* analysis also provides the gene that is potentially driving this co-expression alteration (what in the package function is described as a DCG). Further information about the method is available in the original article (Yang et al., 2013).

For P-value adjustment, the FDR method was performed, hence adjusted P-Values of < 0.05 were included. For the TFs analysis, we incorporated the TRRUST database (Han et al., 2015) in the *DCGL* package, in order to obtain these interactions for *Mus musculus*. TRRUST is a manually curated database that contains transcription factors data for both human and mouse species (Han et al., 2015). The use of this database in *DCGL* package was needed, because the original version of the package only comprises human TFs data (Yang et al., 2013). To accomplish this insertion, we downloaded the

data from TRRUST database, available in the weblink: https://www.grnpedia.org/trrust/downloadnetwork.php, and inserted in R. We emphasize we did not perform any changes in the *DCGL* package algorithms, functions, or methods of analysis. Further specifications about the analysis can be visualized by accessing the R script here used, in the GitHub project https://github.com/thaynewk/thalidomide-co-expression.

Systems biology analyses were processed with Cytoscape v.3.7.2, assembling a network of the differentially co-expressed genes and links obtained in differential co-expression analysis. We inserted the Supplementary Table 8 in Cytoscape and assembled the networks by using the gene-pairs correlation data. Pearson correlation (r) represented the width of the edges. The colors of the edges were chosen arbitrarily to represent same signed (green), differentially signed (orange), and switched opposites (grey) correlations. Deregulator genes (named DCGs in the *DCGL* package) are represented in pink nodes and the other member of the gene-pair is represented as a blue node. Heatmaps were generated using *diffcoexp* R package for Pearson r and *ggplot2* R package for plots, using red to indicate high positive (direct) correlation and blue to indicate negative or inverse correlation; white was used for the gene-pairs that were not biologically correlated (r lower than |±0.5|).
